# Low to Moderate Average Alcohol Consumption and Binge Drinking in Early Pregnancy: Effects on Choice Reaction Time and Information Processing Time in Five-Year-Old Children

**DOI:** 10.1371/journal.pone.0138611

**Published:** 2015-09-18

**Authors:** Tina R. Kilburn, Hanne-Lise Falgreen Eriksen, Mette Underbjerg, Poul Thorsen, Erik Lykke Mortensen, Nils Inge Landrø, Leiv S. Bakketeig, Jakob Grove, Claus Sværke, Ulrik Schiøler Kesmodel

**Affiliations:** 1 Department of Research, Child and Adolescent Psychiatry, Aarhus University Hospital, Risskov, Denmark; 2 Department of Public Health, University of Copenhagen, Copenhagen, Denmark; 3 Vejlefjord Rehabilitation Center, Stouby, Denmark; 4 Department of Obstetrics and Gynaecology, Lillebælt Hospital, Kolding, Denmark; 5 Center for Healthy Aging, University of Copenhagen, Copenhagen, Denmark; 6 Clinical Neuroscience Research Group, Department of Psychology, University of Oslo, Oslo, Norway; 7 National Institute of Public Health, University of Oslo, Oslo, Norway; 8 Department of Biomedicine, Aarhus University, Aarhus, Denmark; 9 Bioinformatics Research Centre, Aarhus University, Aarhus, Denmark; 10 Clinical Epidemiology, Aarhus University, Aarhus, Denmark; 11 Department of Obstetrics and Gynaecology, Aarhus University Hospital, Aarhus, Denmark; 12 Department of Clinical Medicine, Aarhus University, Aarhus, Denmark; Mayo Clinic, UNITED STATES

## Abstract

**Background:**

Deficits in information processing may be a core deficit after fetal alcohol exposure. This study was designed to investigate the possible effects of weekly low to moderate maternal alcohol consumption and binge drinking episodes in early pregnancy on choice reaction time (CRT) and information processing time (IPT) in young children.

**Method:**

Participants were sampled based on maternal alcohol consumption during pregnancy. At the age of 60–64 months, 1,333 children were administered a modified version of the Sternberg paradigm to assess CRT and IPT. In addition, a test of general intelligence (WPPSI-R) was administered.

**Results:**

Adjusted for a wide range of potential confounders, this study showed no significant effects of average weekly maternal alcohol consumption during pregnancy on CRT or IPT. There was, however, an indication of slower CRT associated with binge drinking episodes in gestational weeks 1–4.

**Conclusion:**

This study observed no significant effects of average weekly maternal alcohol consumption during pregnancy on CRT or IPT as assessed by the Sternberg paradigm. However, there were some indications of CRT being associated with binge drinking during very early pregnancy. Further large-scale studies are needed to investigate effects of different patterns of maternal alcohol consumption on basic cognitive processes in offspring.

## Introduction

It is well established that heavy maternal alcohol consumption during pregnancy is associated with fetal alcohol syndrome (FAS)[1] and with a number of cognitive deficits and behavioral dysfunctions reflecting central nervous system dysfunction.[2,3] It is less clear, however, whether lower intake levels are also associated with negative outcomes of pregnancy and offspring development.[4,5] Thus, a large Danish study of 5-year old children showed little consistent evidence of effects of maternal consumption of 1–8 drinks per week on intelligence,[6,7] attention, [7,8] executive functions,[7,9] or behavioral problems.[10] These negative findings are corroborated by a lack of consistent effects in a large scale British study.[11–13]

Animal models suggest that the effects of alcohol on brain growth and development primarily depend upon the level of blood alcohol concentration.[14] Thus, it is relevant to distinguish between intake patterns and to focus on sporadic intake of larger quantities of alcohol, i.e. binge drinking [15] (in humans usually defined as 5 or more drinks per occasion) when assessing potential damages caused by prenatal alcohol exposure. In one human study, prenatal exposure to binge drinking was found to be associated with learning disabilities, deficits in attention and memory, and inflexible approach to problem solving at age 7½ years.[16,17] A recent meta-analysis showed a significant detrimental effect of binge drinking on child cognition when all studies were included, but not when assessing high quality studies only.[18] The recent Danish studies have, however, showed little evidence of effects of binge drinking on a broad range of neurocognitive and neurobehavioral outcomes.[6–10,19]

Since the drinking habits of the large majority of women are characterized by a low regular intake or occasional binge drinking episodes rather than regular heavy consumption, it is important to investigate whether low average consumption and binge drinking affect complex or more basic cognitive functions. Many of the studies finding no effects on offspring cognition have analyzed complex outcomes, such as intelligence or executive functions, and it is possible that more basic cognitive functions may be more sensitive to the effects of prenatal alcohol exposure.

Animal studies have shown that alcohol exposure in utero may target the glial cells and thus disrupt myelination in the developing brain,[20] and human studies suggest that alcohol intake by the mother during the first vulnerable period have an effect on the general development of the nervous system, including myelination.[20,21] Abnormal myelination could be linked with disruption of primary cognitive processes including information processing time (IPT), i.e. the time it takes to process important information in short term memory (STM).[22] IPT has been suggested as a common mechanism underlying cognitive deficits in prenatally alcohol exposed children.[23–25] It may play an important role in higher cognitive functions such as memory and attention and may also be related to general intelligence.[26] In addition, IPT is conceptualized within the broader framework of executive control because it is based on processes of preparation and inhibition of inappropriate responses.[27] Thus, if a child’s IPT is impaired, this may compromise the child’s social and academic development and function in everyday life.

The aim of the present study was to examine individual differences in choice reaction time (CRT) and IPT in relation to low to moderate average alcohol intake and binge drinking during pregnancy by using a version of Sternberg’s paradigm [28] specifically adapted to young children. In the Sternberg paradigm, CRT increases as a function of the number of items to be searched through in STM. The slope reflecting this increase yields a direct measure of speed, which is independent of variations in perceptual skills, decision strategies and response abilities. A steeper slope would thus indicate less efficient processing, and based on findings in previous studies, it was hypothesized that prenatal alcohol exposure is associated with both longer CRT and steeper slopes, indicating longer information processing time (IPT).

## Methods

### Study sample

The Lifestyle During Pregnancy Study (LDPS) has been described in detail elsewhere, including the oversampling of women with moderate to high alcohol intake and binge drinking. [6,8,9,10,19,29,30,31] Briefly, the study is a prospective follow-up, based on a sample from the Danish National Birth Cohort (DNBC consisting of 101,042 women and their children. Women in the DNBC were recruited from 1997 to 2003 at their first antenatal visit to a general practitioner and represent 60% of those invited and approximately 30% of all pregnant women in Denmark in the enrollment period. Based on their alcohol intake during pregnancy, a total of 3,189 women were sampled from the DNBC and invited to participate in the LDPS between 2003 and 2008.[29] Of these women, 1,628 responded positively to the invitation to participate in a 5-year follow-up.

Exclusion criteria for the LDPS were inability to speak Danish, inability to complete the cognitive tests because of impaired hearing or vision, multiple pregnancies, and congenital diseases likely to cause mental retardation (e.g. Down’s syndrome).

### The five-year follow-up

At the age range of 60–64 months, the selected children participated in a comprehensive neuropsychological assessment. In addition to standardized tests for children the test battery included interviews and standardized questionnaires completed by parents and kindergarten teachers.[29]

The follow-up included assessment of intelligence with subtests from the Wechsler Primary and Preschool Scales of Intelligence—Revised (WPPSI-R).[32] IQ was pro-rated using three verbal subtests (Information, Vocabulary, and Arithmetic) and three performance subtests (Object Assembly, Geometric Design and Block Design). Since Danish norms for the WPPSI-R were not available at the time of the study, Swedish norms were used to derive IQs.

As described in previously publications from the LDPS [6,8] testing took place in four major cities of Denmark (Copenhagen, Odense, Aalborg, and Aarhus). Test procedures were standardized in detail and carried out by ten trained psychologists blinded to the children’s exposure status. Tester differences were taken into account by the inclusion of indicator variables in the statistical analyses.

### Sternberg Paradigm

The LDPS developed computer-administered Sternberg paradigm for children was used to assess CRT and IPT.[28] The stimulus presentation software *E-prime* measured the children’s reaction time in milliseconds. On a computer screen placed approximately 40 cm from the child, a trial consisting of images divided into rows of 2 and 3 was shown for 1.50 seconds, followed—after a one second break—by a single image (probe) that remained on the screen until the child responded. A small star, warning the child before each trial, was shown in the middle of the screen for 1 second before the next set of images was presented. The images comprised nine black and white drawings of wild animals: bear, elephant, monkey, lion, kangaroo, rhinoceros, parrot, tortoise and walrus.

The child was told to press the green ‘M’ key for ‘yes’ if the probe was in the previously presented set of pictures or the red ‘X’ key for ‘no’ if it was not (the adjusted keyboard had only these two response keys). For correct responses, a happy Calvin (from the cartoon ‘Calvin and Hobbes’) was presented on the screen shouting ‘Yippee’ and for incorrect responses he would appear in a disappointed manner reacting with an ‘O-oh’. The children were given up to 20 practice trials before starting the actual task.

The experimental trials were divided into 4 blocks of 9, accumulating 36 trials in all. There was a break between blocks for as long as the child needed—usually between 10 seconds to 1 minute, and during this break a cartoon figure, showing a man taking a nap, would be shown on the computer screen. The 36 trials comprised 9 trials for each four basic conditions: 2 images with the probe present, 2 images without the probe, 3 images with the probe present, and 3 images without the probe. Eleven children who had not completed at least two trials for each of the four basic conditions were excluded from data analysis.

### Exposure

Information on alcohol intake during pregnancy was derived from the first prenatal DNBC interview [33] which was conducted at a median gestational age of 17 weeks (range 7–39). The total number of weekly drinks was calculated by asking the women about the average number of beers, glasses of wine and glasses of spirits they currently consumed during a week. This procedure has been shown to yield reliable information among pregnant Danish women.[34] The definition of a drink followed the definition from the Danish Health and Medicines Authority, one standard drink being equal to 12 grams of pure alcohol. All mothers were sampled in strata defined by their average alcohol intake (0, 1–4, 5–8, ≥9 drinks per week) and timing of binge episodes, defined as ≥ 5 drinks on one occasion in gestational weeks 1–2, 3–4, 5–8, ≥9.[29] As described previously [6,8] the higher exposure categories were oversampled in an effort to ensure that all exposure categories included enough children to attain sufficient statistical power. For each sample stratum the sampling probability was computed as the ratio between the number of sampled women and the total number of women available in the corresponding DNBC stratum.

### Covariates

As described previously [6,8,30] the following covariates were obtained in the prenatal interview and subsequently coded as follows: parity (0, 1, ≥2); prenatal maternal smoking (yes/no); maternal pre-pregnancy BMI (weight in kg/(height in m)^2^). At the time of the 5-year follow-up, the following variables were recorded: length of parental education in years (the average educational length for the two parents or length of maternal education if information on the father was unavailable); marital status (single either at prenatal interview or at follow-up/married or cohabitating at both); postnatal parental smoking (yes, if at least one of the parents smoked in the home/no if otherwise); child health status (presence of any medical conditions or regular medications that might influence test performance: epilepsy, syndromes such as Morpheus syndrome, Neurofibromatosis Recklinghausen, congenital toxoplasmosis and myxedema, medicines for asthma and allergy, ADHD [methylphenidate], epilepsy and respiratory conditions); dichotomized family/home environment index (presence of two or more of the following seven adverse conditions: not living with a biological parent, changes in caregiver, day care for more than 8 hours/day before age 3, ≥14 days outside of home, breakfast irregularity (whether or not the child is served breakfast every day), maternal depression, and parental alcohol use above the maximum recommended level by the Danish Health and Medicines Authority of 14 drinks per week for women or 21 for men); hearing ability (impaired/not impaired); vision ability (impaired/not impaired) (Table 1).

**Table 1:** Sample characteristics across levels of average maternal alcohol intake in pregnancy.

	Average number of drinks per week				
	0	1–4	5–8	≥ 9 ^1^	Total
Number of participants	629	549	138	17	1333
Sampling fraction*(Median*, *10th/90th pctile)*	8.0(1.5/49.6)	5.5(1.2/22.8)	65.6(34.3/76.5)	95.0(57.9/95.0)	9.7(1.5/49.6)
Timing of interview*(Gestational week)*	16.0(13.0/23.0)	17.0(13.0/24.0)	17.0(13.0/24.0)	18.0(12.0/28.0)	17.0(13.0/24.0)
Maternal age, years*(Mean ± SD)*	29.8 ± 4.3	31.5 ± 4.0	32.8 ± 4.5	35.4 ± 5.0	30.9 ± 4.3
Parity					
*0 (%)*	56.4	48.3	29.7	11.8	49.7
*1 (%)*	30.7	32.8	44.2	41.2	33.1
*2+ (%)*	12.9	18.9	26.1	47.1	17.2
Maternal BMI, kg/m^2^ *(Median*, *10/90 pctile)*	22.6(19.5/29.1)	22.6(19.7/28.4)	22.4(19.6/28.1)	21.6(18.0/29.4)	22.5(19.6/28.7)
Maternal marital status ^2^ *Single (%)*	12.0	10.8	11.7	5.9	11.4
Parental education, years*(Median*, *10/90 pctile)*	13.0(11.0/16.0)	13.0(11.0/16.0)	13.0(11.0/17.0)	12.5(11.0/17.0)	13.0(11.0/16.0)
Family/home index*Suboptimal* ^3^ *(%)*	16.9	15.5	26.1	35.3	17.5
Maternal IQ *(Mean ± SD)*	99.8 ± 15.0	102.0 ± 14.4	100.9 ± 15.9	101.3 ± 14.9	100.8 ± 14.8
Maternal smoking in pregnancy*Smokers (%)*	30.5	25.9	39.9	64.7	30.0
Postnatal parental smoking *Smokers (%)*	29.0	29.3	37.7	58.8	30.4
Maternal binge drinking in pregnancy ^4^ *(%)*	66.8	77.8	58.0	41.2	70.1
Child characteristics					
Sex *Male (%)*	47.5	53.9	54.3	41.2	50.8
Age at testing, years*(Median*, *10/90 pctile)*	5.2(5.1/5.3)	5.2(5.1/5.3)	5.2(5.1/5.3)	5.3(5.2/5.3)	5.2(5.1/5.3)
Birth weight, grams	3608.9± 525.1	3635.7± 488.5	3575.0± 496.3	3351.5± 596.5	3613.1± 509.0
Gestational age, days*(Median*, *10/90 pctile)*	281.0(267.0/293.0)	282.0(269.0/293.0)	282.5(268.0/292.0)	275.0(256.0/294.0)	282.0(268.0/293.0)
Health status *Condition/medicine* ^5^ *(%)*	3.3	2.9	5.8	5.9	3.5
Hearing abilities*Normal (%)*	96.5	94.3	95.7	88.2	95.4
Vision abilities*Normal (%)*	97.3	98.0	95.7	94.1	97.4

^1^. Range 9–14 drinks/week.

^2^. Single, if single either in pregnancy or at follow-up (60–64 months postpartum).

^3^. Defined as a score on at least 2 of the following items: single parent household; changes in care giving; day care >8 hrs/day before age 3; 14+ days away from home; irregular breakfast meals; maternal depression; high maternal or paternal alcohol use.

^4^. Defined as intake of 5 drinks or more at one occasion.

^5^. Medical conditions or regular medications that may influence test performance.

Maternal IQ was assessed at the follow-up examination: Two verbal subtests (Information and Vocabulary) from the Wechsler Adult Intelligence Scale (WAIS)[35] were used to assess verbal IQ, and the Raven’s Standard Progressive Matrices [36] provided non-verbal IQ. Raw scores of each test were standardized based on the results from the full sample and weighted equally in a combined score that was re-standardized to an IQ scale with a mean of 100 and an SD of 15.

Maternal age was obtained directly from the unique Danish personal identification number, as was sex of child and age of child at testing. Birth weight (g) and gestational age (days) were obtained from the Danish Birth Registry.

All core and potential confounders were defined a priori as described elsewhere [8,30]

### Statistical analysis

To minimize the influence of unrealistically slow and fast reactions, outlier definitions were determined prior to initiation of the protocol and were excluded before analyses using the following criteria: The reaction time was considered to be an outlier if it was less than 200 ms (50 reaction times out of 45,229), or if it (calculated on the log-scale) was larger than the 75^th^-percentile plus three times the width of the interquartile range (219 reaction times). Both the percentile and the interquartile ranges were computed on the whole sample stratified on 2 and 3 images and on the presence or absence of the probe.

Like most IPT studies, we only analyzed reaction times with correct answers since incorrect answers may be the result of loss of attention, concentration, or willingness to continue participation.[16,37,38]

All statistical analyses were conducted in Stata 11 (StataCorp LP, College Station, Texas) and weighted by sampling probabilities with robust variance estimation. All statistical tests were two-sided and declared significant at the 5% level.

Sufficient Sternberg data were available for 1,333 children (82% of the 1,628 tested). The test was not administered to 172 children, too few test data were available for 11 children, and hardware failure resulted in the exclusion of further 112 children.

A total of 1,249 children had complete data for all 36 trials on the Sternberg paradigm. For the remaining 84 children the number of completed trials ranged from 8 to 33, and the number of missing values for the covariates ranged from 0 to 27. Missing values were imputed based on two strategies: A dedicated model for imputation where missing values were modeled from the other variables considered to be most predictive of the variable with missing values (specific equations are available upon request), and a black-box strategy with all outcomes, exposures, and covariates used to predict missing values. Regardless of imputation strategy, the main conclusions were unaffected, and the point estimates of the exposure parameters never differed by more than 18% relative to their standard error [39]. Essentially the same results were obtained when the strategy of complete case analysis was used, and only results based on the dedicated imputation strategy will be reported.

The primary outcome measures were 1) CRT: choice reaction time based on log-transformed (natural logarithm) reaction times, and 2) IPT: based on the slope in average log-transformed choice reaction times for 3 images minus average for 2 images for trials with correct answers. The slope was also computed for conditions corresponding to whether or not the probe was present. We analyzed CRT and IPT for average number of drinks per week (0, 1–4, 5–8, ≥ 9) and for the following binge drinking patterns: binge drinking (yes/no); number of binge episodes (0, 1, 2, ≥3); and timing/gestational week of binge drinking episodes (1–2, 3–4, 5–8, ≥9, or multiple episodes (≥2 of the time periods).

In supplementary analyses, we analyzed an additional outcome measure, the proportion of correct answers based on all trials with 2 images and 3 images, respectively. We also analyzed potential interactions between average alcohol consumption and binge drinking and interactions of these factors with smoking, sex of child, and parental education.

As in previous analysis of the LDPS-data [8,30] parental education, maternal IQ, prenatal maternal smoking, the child’s age at testing, child’s sex, and tester were considered core confounders, while the full model also controlled for maternal age, pre-pregnancy BMI, parity, marital status, home environment, postnatal parental smoking, child’s health status, and indicators for hearing and vision impairment. In the full models prenatal maternal binge drinking (coded yes/no) was included as potential confounder in analyses of average number of drinks per weeks, while maternal average number of drinks per week during pregnancy (coded 0, 1–4, 5–8, ≥ 9 drinks per week) was included as in the analyses of effects of binge drinking.

Birth weight and gestational age were considered potential mediators of the effects of alcohol exposure on test performance. Consequently, these variables were not included in the main analysis, but included in separate models evaluating potential mediation.

### Details of ethics approval

The study was approved by the Danish National Birth Cohort Board of Directors, the Danish National Birth Cohort Steering Committee, the Regional Ethics Committee (Videnskabsetiske komité for Aarhus Amt (journalno. 20020227), the Danish Data Protection Agency (Datatilsynet, (journalno. 2002-41-2166), and the Institutional Review Board at the Centers for Disease Control and Prevention, Atlanta, USA.

Written informed consent was obtained from the next of kin or guardians on behalf of the children enrolled in the LDPS.

## Results

Women with higher weekly intake tended to be older, and there were higher rates of suboptimal home environment and smoking among women in the 5–8 and ≥ 9 exposure categories. Binge drinking episodes were most common among women consuming 1–4 drinks per week, but a substantial number of women reported binge drinking episodes although they reported no average weekly alcohol intake. Otherwise, no prominent differences between non-exposed and exposed participants were seen (Table 1). Mothers to participating children without a score on the Sternberg paradigm were more often single mothers and smokers, and there were relatively more boys among the children without a score (Table 2). An overall accuracy rate of 77% was obtained, indicating that the children could follow instructions and understand the Sternberg task. Ignoring the 112 children whose results were lost due to hardware failure, 172 out of 1,516 children were not administered the task and 11 were unable to complete the task, corresponding to about 12% of the children. Non-administration and task failure might have been related to the comprehensive and tiring test battery, but more importantly to both the child’s cognitive functioning and maternal alcohol consumption, and in fact the mean full scale IQ of the 172+11 children was 11 IQ points lower than the mean of the 1,333 children with a Sternberg score (p <0.001).

**Table 2:** Maternal and child characteristics of participants and non-participants.

	Sternberg sample	No Sternberg score	Non-participants	Total
Number of participants	1,333	295	1,561	3,189
Sampling fraction	9.7(1.5/49.6)	9.7(1.2/49.6)	8.0(1.2/49.6)	8.0(1.5/49.6)
Timing of interview *(Gestational week)*	17.0(13.0/24.0)	17.0(13.0/23.0)	17.0(13.0/24.0)	17.0(13.0/24.0)
Maternal characteristics				
Age, years *(Mean ± SD)*	30.9 ± 4.3	30.7 ± 4.6	30.3 ± 4.6	30.6 ± 4.5
Prenatal marital status*Single (%)*	2.4	5.8	2.9	3.0
Parity				
*0 (%)*	49.7	51.5	49.1	49.5
*1 (%)*	33.1	28.1	33.2	32.7
*2 (%)*	17.2	20.3	17.7	17.7
BMI, kg/m^2^ *(Median*, *10/90 pctile)*	22.5(19.6/28.7)	23.0(19.6/28.4)	23.0(19.4/29.7)	22.8(19.5/29.1)
Smoking in pregnancy *Smokers (%)*	30.0	37.6	34.5	32.9
Binge drinking in pregnancy ^1^ *(%)*	70.1	67.5	65.3	67.5
Alcohol intake in pregnancy, drinks/week *(Mean ± SD)*				
*0*	47.2	43.7	50.4	48.4
*1–4*	41.2	42.7	38.9	40.2
*5–8*	10.4	12.5	10.1	10.4
*≥ 9* ^2^	1.3	1.0	0.6	0.9
Child characteristics				
Sex*Male (%)*	50.8	57.3	51.2	51.6
Birth weight, grams *(Mean ± SD)*	3613.1 ± 509.0	3551.2 ± 545.2	3546.4 ± 555.7	3574.7 ± 536.5
Gestational age at birth, days *(Median*, *10/90 pctile)*	282.0(268.0/293.0)	280.0(266.0/293.0)	281.0(264.0/293.0)	281.0(266.0/293.0)

^1^. Defined as intake of 5 drinks or more at one occasion

^2^. Range 9–14

The children completing the Sternberg test performed consistently. Thus, the reliability of CRT was estimated to 0.78 and 0.76 for two and three pictures respectively and 0.85 for the total reaction time score based on correlations between two parts of the 36 trials (results from block 1 and 3 correlated with results from block 2 and 4). For the total test the correlation between 2 and 3 images was 0.80, suggesting that most children tended to be slow or fast both on trials with 2 and 3 images.

### Average weekly intake

Analyses of choice reaction time (Table 3) showed non-significant results for all exposure categories, with shorter average reaction times for the 1–4 category compared with the reference, while the mean CRT of the 5–8 category was non-significantly longer after adjustment for core and potential confounders. Mean reaction times were longest for the ≥ 9 category, with a wide 95% CI as a consequence of the small number of observations.

**Table 3:** Associations between average maternal alcohol intake in pregnancy and choice reaction time (CRT) on the Sternberg task ^1^.

	Crude			Adjusted for core confounders ^2^		Adjusted for potential confounders ^3^	
Average no. drinks/week in pregnancy	Mean CRT	Mean difference	95% CI	Mean difference	95% CI	Mean difference	95% CI
0	6.29	Reference	-	Reference	-	Reference	-
1–4	6.26	-0.03	[-0.06; 0.00]	-0.02	[-0.06; 0.01]	-0.02	[-0.06; 0.01]
5–8	6.27	-0.02	[-0.08; 0.05]	0.01	[-0.06; 0.07]	-0.00	[-0.06; 0.06]
≥ 9	6.35	0.06	[-0.05; 0.18]	0.09	[-0.03; 0.20]	0.10	[-0.02; 0.22]
*p value* ^4^		*0*.*21*		*0*.*21*		*0*.*15*	

^1^. Sternberg version for children.

^2^. Parental education, maternal IQ, prenatal maternal smoking, the child’s sex and age, and tester.

^3^. Parental education, maternal IQ, prenatal maternal smoking and binge drinking, maternal age, parity, prenatal and postnatal marital status, postnatal parental smoking, maternal pre-pregnancy BMI, the child’s sex and age, health status, hearing and vision on the day of testing, family/home environment, and tester.

^4^. *P* value for the hypothesis of no difference in attention scores across levels of average alcohol intake.

Analyses of slope or IPT in relation to average alcohol consumption showed no significant differences between the exposed categories and the reference category. Table 4 shows that this was the case for both the analyses based on all reaction times with correct answer as well as for the separate analyses of trials with probe present and trials without probe present.

**Table 4:** Associations between average maternal alcohol intake in pregnancy and slope ^1^ on the Sternberg task ^2^.

	Crude			Adjusted for core confounders ^3^		Adjusted for potential confounders ^4^	
Average no. drinks/week inpregnancy	Slope	Mean difference	95% CI	Mean difference	95% CI	Mean difference	95% CI
Overall slope							
0	0.08	Reference	^-^	Reference	-	Reference	-
1–4	0.11	0.03	[-0.01; 0.06]	0.03	[-0.00; 0.06]	0.03	[-0.00; 0.06]
5–8	0.10	0.02	[-0.06; 0.11]	0.04	[-0.04; 0.11]	0.04	[-0.04; 0.12]
≥ 9	0.08	0.00	[-0.08; 0.08]	0.02	[-0.07; 0.10]	0.03	[-0.06; 0.12]
*p value* ^5^		*0*.*45*		*0*.*36*		*0*.*34*	
Slope for trials with probe present							
0	0.07	Reference	-	Reference	-	Reference	-
1–4	0.09	0.02	[-0.02; 0.06]	0.02	[-0.02; 0.06]	0.02	[-0.02; 0.06]
5–8	0.08	0.00	[-0.12; 0.13]	0.02	[-0.10; 0.15]	0.03	[-0.09; 0.16]
≥ 9	0.03	-0.04	[-0.17;0.08]	-0.03	[-0.16; 0.11]	-0.01	[-0.14; 0.13]
*p value*		*0*.*71*		*0*.*79*		*0*.*80*	
Slope for trials without probe present							
0	-0.04	Reference	-	Reference	-	Reference	-
1–4	0.01	0.05	[-0.00; 0.09]	0.04	[-0.01; 0.09]	0.04	[-0.01; 0.09]
5–8	0.00	0.04	[-0.06; 0.14]	0.04	[-0.06; 0.15]	0.04	[-0.06; 0.15]
≥ 9	-0.05	-0.01	[-0.13; 0.11]	-0.00	[-0.13; 0.12]	0.02	[-0.12; 0.16]
*p value*		*0*.*24*		*0*.*36*		*0*.*38*	

^1^. Slope calculated as the difference between the mean log transformed reaction times for the trials with 2 and 3 images respectively (correct answers only).

^2^. Sternberg version for children.

^3^. Parental education, maternal IQ, prenatal maternal smoking, the child’s sex and age, and tester.

^4^. Parental education, maternal IQ, prenatal maternal smoking and binge drinking, maternal age, parity, prenatal and postnatal marital status, postnatal parental smoking, maternal pre-pregnancy BMI, the child’s sex and age, health status, hearing and vision on the day of testing, family/home environment, and tester.

^5^. *P* value for the hypothesis of no difference in attention scores across levels of average alcohol intake.

### Binge drinking

The unadjusted results for CRT (Table 5) showed insignificantly longer reaction times for the dichotomous binge variable, for one binge episode, and for binge drinking in gestational weeks 1–2 compared to no binge episodes. After adjustment for core confounders, the effect of one binge episode was statistically significant (mean difference on log scale = 0.03, 95% CI = 0.00; 0.06, corresponding to an increase in reaction time on normal scale of 3.5% (95% CI = 0.4%; 6.6%) as was the effect of binge drinking in gestational weeks 1–2 (increase on normal scale = 4.8%, 95% CI = 1.0; 8.7); these results did not change when adjusting for all confounders. No similar trends were observed for the other binge categories, their estimated effects being insignificant and close to zero.

**Table 5:** Associations between maternal binge drinking in pregnancy and outcomes on choice reaction time (CRT) on the Sternberg task ^1^, Denmark 2003–2008.

	Crude			Adjusted for core confounders ^2^		Adjusted for potential confounders ^3^	
	Mean CRT	Mean difference	95% CI	Mean difference	95% CI	Mean difference	95% CI
Binge drinking in pregnancy							
No	6.26	Reference	-	Reference	-	Reference	-
Yes	6.28	0.02	[-0.01; 0.05]	0.03	[-0.00; 0.05]	0.03	[-0.00; 0.05]
*p-value* ^4^	*0*.*22*			*0*.*08*		*0*.*08*	
Number of binge drinking episodes in pregnancy							
0	6.27	Reference	-	Reference	-	Reference	-
1	6.30	0.03	[0.00; 0.06]	0.03	[0.00; 0.06]	0.04	[0.00; 0.07]
2	6.27	-0.00	[-0.05; 0.04]	0.01	[-0.03; 0.05]	0.01	[-0.04; 0.05]
≥3 ^5^	6.26	-0.01	[-0.06; 0.04]	0.01	[-0.05; 0.06]	0.01	[-0.05; 0.06]
*p-value*	*0*.*11*			*0*.*16*		*0*.*14*	
Timing of binge drinking episodes in pregnancy (gestational week)							
No binge drinking	6.27	Reference	-	Reference	-	Reference	-
1–2	6.31	0.04	[0.00; 0.08]	0.05	[0.01; 0.08]	0.04	[0.01; 0.08]
3–4	6.30	0.03	[-0.01; 0.07]	0.04	[-0.00; 0.07]	0.04	[0.00; 0.08]
5–8	6.26	-0.01	[-0.04; 0.03]	-0.01	[-0.05; 0.03]	-0.01	[-0.05; 0.04]
≥ 9	6.27	0.00	[-0.03; 0.04]	0.00	[-0.04; 0.04]	0.02	[-0.03; 0.06]
Multiple episodes	6.26	-0.01	[-0.05; 0.04]	0.01	[-0.04; 0.06]	0.00	[-0.04; 0.05]
*p-value*	*0*.*16*			*0*.*05*		*0*.*06*	

^1^. Sternberg version for children.

^2^. Parental education, maternal IQ, prenatal maternal smoking, age at testing, sex of child, and tester.

^3^. Parental education, maternal IQ, prenatal maternal smoking, prenatal maternal average alcohol intake, maternal age, parity, prenatal and postnatal marital status, postnatal parental smoking, maternal pre-pregnancy BMI, sex of child, age at testing, health status, hearing and vision on the day of testing and family/home environment.

^4^. *P*-value for the hypothesis of no difference in mean attention scores across levels of average alcohol intake.

^5^. Range: 3–12 episodes.

To assess whether the significant effects on CRT reflected an effect on child IQ, the WPPSI-R Full Scale IQ was added to the full model (results not shown), but this did not change the results.

Analyses of the associations between maternal binge drinking in pregnancy and IPT showed no association between any of the binge categories and slope in unadjusted or adjusted analyses, all estimates being non-significant and very close to zero (Table 6).

**Table 6:** Associations between maternal binge drinking in pregnancy and slope ^1^ on the Sternberg task^2^, Denmark 2003–2008.

	Crude			Adjusted for core confounders ^3^		Adjusted for potential confounders ^4^	
	Mean slope^4^	Mean difference	95% CI	Mean difference	95% CI	Mean difference	95% CI
Binge drinking in pregnancy							
No	0.09	Reference	-	Reference	-	Reference	-
Yes	0.09	0.00	[-0.02; 0.03]	0.00	[-0.02; 0.03]	0.00	[-0.03; 0.02]
*p-value* ^5^	*0*.*88*			*0*.*81*		*0*.*93*	
Number of binge drinking episodes in pregnancy							
0	0.09	Reference	-	Reference	-	Reference	-
1	0.10	0.01	[-0.02; 0.04]	0.01	[-0.02; 0.04]	0.01	[-0.02; 0.03]
2	0.08	-0.01	[-0.05; 0.03]	-0.01	[-0.05; 0.03]	-0.01	[-0.05; 0.03]
≥3 ^6^	0.07	-0.02	[-0.07; 0.03]	-0.01	[-0.07; 0.04]	-0.02	[-0.07; 0.03]
*p-value*	*0*.*54*			*0*.*66*		*0*.*62*	
Timing of binge drinking episodes in pregnancy (gestational week)							
No binge drinking	0.09	Reference	-	Reference	-	Reference	-
1–2	0.10	0.01	[-0.02; 0.04]	0.01	[-0.02; 0.04]	0.01	[-0.03; 0.04]
3–4	0.09	-0.00	[-0.04; 0.03]	-0.00	[-0.04; 0.03]	-0.01	[-0.04; 0.03]
5–8	0.10	0.01	[-0.03; 0.04]	0.01	[-0.03; 0.04]	0.00	[-0.03; 0.04]
≥ 9	0.10	0.00	[-0.03; 0.04]	0.01	[-0.02; 0.04]	0.01	[-0.03; 0.04]
Multiple episodes	0.09	-0.00	[-0.04; 0.03]	0.00	[-0.04; 0.04]	-0.00	[-0.04; 0.04]
*p-value*	*0*.*97*			*0*.*98*		*0*.*96*	

^1^. Slope calculated as the difference between the mean log transformed reaction times for the trials with 2 and 3 images respectively (correct answers only).

^2^. Sternberg version for children

^3^. Parental education, maternal IQ, prenatal maternal smoking, age at testing, sex of child, and tester.

^4^. Parental education, maternal IQ, prenatal maternal smoking, prenatal maternal average alcohol intake, maternal age, parity, prenatal and postnatal marital status, postnatal parental smoking, maternal pre-pregnancy BMI, sex of child, age at testing, health status, hearing and vision on the day of testing and family/home environment.

^5^. *P*-value for the hypothesis of no difference in mean attention scores across levels of average alcohol intake.

^6^. Range: 3–12 episodes.

The interaction between average weekly intake and the dichotomous binge drinking variable was significant with slower CRT associated with a combination of ≥ 9 drinks per week and at least one binge episode. No other significant interactions were observed of maternal average weekly intake with binge drinking or of average consumption and binge drinking with smoking, sex of child, or parental education respectively (results not shown).

To assess whether the significant effects on CRT reflected an effect on child IQ, the WPPSI-R Full Scale IQ was added to the full model (results not shown), but this did not change the results. Furthermore, including birth weight and gestational age in the models for CRT did not change the results, suggesting that the observed effects of alcohol exposure are not mediated by these early growth factors.

### Correct Answers

The overall results for correct answers for 2 and 3 image trials respectively showed small and insignificant differences for average weekly intake as well as binge drinking (results not shown). In analyses of trials with 2 images, all estimates were negative for the 5–8 and ≥9 drinks per week categories. It was more negative for the ≥9 drinks per week category (adj. mean difference = -1.02 ms, 95% CI = -2.34; 0.30), but only significant for 5–8 drinks per week in the core confounder adjusted analyses (mean difference = -0.80, 95% CI = -1.58; -0.01). However, we did not observe a similar trend for trials with 3 images (results not shown).

## Discussion

In this study we analyzed the effects of average low to moderate alcohol consumption and binge drinking episodes in early pregnancy on offspring choice reaction time and information processing time measured with a Sternberg paradigm modified to fit the ability of 5-year-old children. Adjusted for a wide range of potential confounders, no significant associations of child IPT with maternal average weekly alcohol consumption or maternal binge drinking were observed. However, there were some indications that CRT was associated with specific patterns of binge drinking during pregnancy, and that the number of correct answers might be related to maternal average weekly alcohol consumption.

The overall negative findings of this study is in line with a number of previous studies based on the LDPS consistently reporting non-significant associations of low to moderate weekly consumption and binge drinking episodes with complex outcomes in offspring, such as intelligence, attention, executive functions, psychomotor functions, and general behavior.[7–10,19,30,39] This study adds important information regarding potential effects of low maternal alcohol consumption and binge drinking on more basic cognitive functions which are the basis for higher cognitive functions.

Specific effects on IPT and CRT following heavy prenatal alcohol exposure associated with FAS have previously been reported for both heavy doses associated with FAS [16,40–42] and for lower consumption levels comparable to those investigated in the present study. Thus one study reported slower IPT on a hand coordination task following low exposure levels at age 16–19 years (mean exposure = 0.75/0.26/0.32 units/day for each trimester; n = 320).[43] Streissguth et al.[42,44,45] reported a linear dose-response relation between log average oz alcohol/day and reaction time at age 4, an effect still present at a 14-year follow-up.

On the other hand, Burden et al.[16] examined 337 black children at the age of 7.5 years using a task built on Sternberg’s paradigm and found no significant relation between a daily maternal alcohol intake of >1 drink/day during pregnancy and IPT. They did, however, find a non-significant trend of slower overall CRT related to alcohol consumption, but no relation between exposure and accuracy, or exposure and simple reaction time (i.e., reaction time not involving short-term memory search or response selection). Likewise, no differences in simple reaction time were observed between a group of children with FAS compared with a group of non-FAS alcohol exposed children and a group of unexposed children.[46] The sample size of this study was, however, small, with 20 children or less in each exposure group.

In conclusion, while some previous studies reported effects on IPT of prenatal alcohol exposure even in low to moderate doses, we did not find evidence in our sample of effects of low to moderate average maternal consumption. There was, however, an indication of maternal binge drinking in pregnancy being associated with longer CRT.

### Limitations and strengths

An important methodological issue is our assessment of CRT and IPT since information processing time, based on the original Sternberg paradigm, has not earlier been investigated in 5-year-old children. Our newly developed version of the Sternberg paradigm was adapted to the young age of the children and constructed to make it possible for even very young children to understand and perform the task. Nevertheless, for 12% of the children the task could not be administered or completed and this might be related to both the child’s cognitive functioning and maternal alcohol consumption. In fact, the mean full scale IQ of these children was lower than the mean IQ of the children completing the task, which might indicate that the task demands a certain level of cognition. However, using a similar task the study by Burden et al.[16] observed no effects of maternal alcohol consumption in children only slightly older at 7½ years. Still, possible methodical challenges by using the Sternberg paradigm in 5 year old children should be considered.[28]

It is not obvious why longer reaction time were observed only for one binge episode, and why the effect was not observed for 2 or ≥3 binge episodes if in fact alcohol exposure was the cause. It is possible that the result is a consequence of multiple testing since we did not adjust for the many statistical tests. The association could also be a product of misclassification due to underreporting; however, a similar trend towards longer reaction time for a higher number of binge episodes would still be expected. Differential effects by timing of exposure, on the other hand, are biologically plausible to the extent that specific developmental processes active at the time of exposure are targeted, and timing, along with dose and duration, may moderate the manifest effects of in utero alcohol exposure.[47] Even though the development of the central nervous system and the brain continues beyond the second trimester, reaction time, as a basic process presumably related to the more global hard wiring of the brain, could hypothetically be among the processes particularly sensitive to developmental disruption early in fetal life [48]. One study, however, indicated a 5-fold risk of fetal alcohol related disorders with binge exposure in all trimesters compared with exposure only in the first trimester.[49]

The available studies differ substantially from our study with respect to both sizes and socioeconomic characteristics of the samples, level of alcohol exposure, and confounder control.[16,50] Thus, while most previous studies have focused on a daily alcohol intake, we also focused on the potential risk of intake at lower weekly levels and episodes of binge drinking. In the LDPS sample, maternal IQ and parental education have previously been demonstrated to be perhaps the most important predictors of the children’s cognitive functioning,[31] and an important strength of our study was the possibility of including maternal IQ and parental education as covariates together with a wide range of potential confounders.

The primary strengths of the present study were the large sample size and the detailed and prospectively collected information on alcohol exposure and potential confounders which minimizes the risk of information bias. The number of highly exposed children in our sample was small, but there were some indications that CRT was associated with specific patterns of binge drinking during pregnancy, and that the number of correct answers was related to maternal average weekly alcohol consumption.

## Conclusion

Low levels of social drinking during pregnancy has previously been considered acceptable in Denmark, and most current evidence on the potentially adverse effects of low to moderate prenatal alcohol exposure suggests that this may not be associated with obvious harm to the fetus.[51] However, subtle long-term effects on cognitive development in offspring, such as effects on information processing speed, can only be detected in studies designed for this specific purpose, as can effects associated with specific intake patterns. Thus, it is essential that more large-scale studies are initiated to illuminate the effects of different patterns of maternal alcohol consumption on basic cognitive processes in offspring.

Although our findings suggest that low amounts of alcohol intake may not seriously harm neurodevelopment, an internationally recognized safe level of consumption during pregnancy has not yet been established. Thus, pregnant women must still be advised to abstain from alcohol during pregnancy if all risk of harmful effects should be avoided.
